# Simulation Study on the Effect of Growth Pressure on Growth Rate of GaN

**DOI:** 10.3390/ma18214941

**Published:** 2025-10-29

**Authors:** Tian Qin, Huidong Yu, Qingbin Liu, Qiubo Li, Zhongxin Wang, Shouzhi Wang, Lihuan Wang, Guodong Wang, Jiaoxian Yu, Zhanguo Qi, Zhengtang Yang, Lei Zhang

**Affiliations:** 1 Institute of Novel Semiconductors, State Key Laboratory of Crystal Material, Shandong University, Jinan 250100, China; qt18678298732@163.com (T.Q.); 202234107@mail.sdu.edu.cn (H.Y.); llhl2x@126.com (Q.L.); wzx2889@163.com (Z.W.); wangsz@sdu.edu.cn (S.W.); zhan_guo_2021@163.com (Z.Q.); yangzt2003@163.com (Z.Y.); 2 Shandong Crystal GaN Semiconductor Co., Ltd., Jinan 250100, China; 3 Key Laboratory of Processing and Testing Technology of Glass & Functional Ceramics of Shandong Province, School of Materials Science and Engineering, Qilu University of Technology (Shandong Academy of Sciences), Jinan 250353, China; 4 Shandong Research Institute of Industrial Technology, Jinan 250100, China

**Keywords:** HVPE, large-size GaN, growth pressure, finite element simulation, single crystal uniformity, growth rate

## Abstract

During the preparation of gallium nitride (GaN) single crystals by Hydride Vapor Phase Epitaxy (HVPE), variations in growth pressure within the reaction chamber can easily lead to a mismatch between vapor transport dynamics and surface reaction processes, thereby affecting crystal growth rate and uniformity. To address this issue, this study established a multi-physics coupled simulation model based on the HVPE equipment structure. By integrating reaction gas flow, heat transfer, chemical reactions, and mass transport mechanisms, systematic finite element analysis was employed to simulate the flow field distribution, thermal field stability, and precursor concentration field evolution within the reaction chamber under different growth pressures (91–141 kPa). The simulation results indicate that, on one hand, the growth rate exhibits a nearly linear increase trend with rising pressure. At lower pressures (<100 kPa), vapor transport is limited, leading to a significant decrease in growth rate, while at higher pressures (>110 kPa), growth uniformity deteriorates. Optimizing the pressure parameter can enhance both the growth rate and thickness uniformity of GaN single crystals, providing a basis for process control in the preparation of high-performance GaN devices.

## 1. Introduction

III-V nitrides, as representative third-generation semiconductor materials, hold significant application prospects in optoelectronics and microelectronics. Consequently, related material growth and device development have garnered widespread attention and achieved substantial progress.

Compared to first- and second-generation semiconductor materials, third-generation semiconductors, primarily represented by silicon carbide (SiC) [1,2,3], aluminum nitride (AlN [4,5,6]), and gallium nitride (GaN) [7,8,9,10,11,12,13,14,15], possess higher breakdown electric fields, electron saturation velocities, thermal conductivities, and wider bandgaps. They are more suitable for developing and manufacturing high-frequency, high-power, radiation-resistant, and corrosion-resistant electronic devices, optoelectronic devices, and light-emitting devices [9,10,11,12,13]. A comparative summary of their key properties, compiled from established references [1,2,3,4,5,6,7,8,9,10,11,12,13,14,15], is provided in Table 1. These materials are thereby gradually becoming the core supporting materials for the third-generation semiconductor industry. Mature device applications require high-quality material preparation technologies as a guarantee. Currently, the preparation of large-size, high-quality GaN materials has become a major factor influencing device performance.

Currently, the main methods for growing GaN single crystals include HVPE, Metal–Organic Chemical Vapor Deposition (MOCVD), Na-flux method, and the ammonothermal method [14,15,16,17,18,19]. Among these, HVPE offers the fastest growth rate, reaching up to 200 μm/h, but the resulting crystals have higher dislocation densities (10^5^–10^6^ cm^−2^) and are prone to cracking and warping, affecting crystal quality. However, its growth conditions are relatively mild, making it one of the most commercially promising methods currently. The advantages of MOCVD [20,21,22,23] include the following: firstly, reactants enter the reaction chamber in gaseous form, allowing precise control over epitaxial material thickness and composition by regulating various gas flows; secondly, the fast gas flow within the reaction chamber enables sharp heterojunction interfaces by changing gas types; thirdly, the grown GaN has fewer impurities and high crystal quality. However, significant challenges remain for MOCVD in growing thick films and bulk materials, such as slow growth rates, large warpage, severe byproduct deposition, and difficulty in continuous long-term operation. The ammonothermal method grows GaN crystals at relatively low temperatures (400–750 °C), yielding high-quality crystals with dislocation densities [24,25,26]. However, the growth rate of GaN by this method is very slow (0.02–1 mm/day), and it requires very high pressures (100–600 MPa), imposing stringent demands on equipment precision and safety, significantly limiting its commercial application. Growth experiments of GaN single crystals on sapphire substrates were conducted by our research group using the HVPE method, investigating the influence of growth temperature on the growth rate and uniformity of large-size GaN single crystals.

Current research on HVPE-GaN still heavily relies on experimental verification [27,28]. Process optimization requires extensive repetitive testing, resulting in limited efficiency and significant resource consumption. Notably, systematic research on the formation mechanisms and theoretical models in the early stages of the growth process has not been established, which lacks theoretical support for understanding the evolution of film quality. However, obvious limitations remain in current simulation studies: On one hand, most studies focus on a single physical field (only analyzing flow field distribution or thermal field stability) and fail to fully consider the multi-physics coupling effects of “reaction gas flow-heat transfer-chemical reactions-mass transport” in the HVPE system. In particular, they overlook the buoyancy-induced migration differences between GaCl and NH_3_ under high-temperature environments due to their molecular mass disparity (approximately 105 g/mol for GaCl and 17 g/mol for NH_3_), making it difficult to accurately describe the actual evolution of the reactant concentration field. On the other hand, existing research primarily concentrates on optimizing parameters such as temperature and gas flow rate, while systematic studies focus on growth pressure—a critical variable that directly affects the matching between vapor transport dynamics and surface reaction rate. Moreover, the generalized models of commercial tools struggle to adapt to the customized structure of vertical HVPE reactors (e.g., embedded gas inlet tubes, dual heating zones, rotating substrates), failing to specifically address the “growth rate—uniformity” balance issue in practical growth processes. Additionally, most simulation studies do not form a closed loop of “simulation prediction—experimental verification,” which limits the practical guiding value of their simulation results.

This study constructs a multi-physics coupled model for HVPE-GaN single crystal growth based on the 2024r2 ANSYS Fluent finite element simulation platform, tailored to the specific structure of the vertical HVPE reactor (cylindrical quartz chamber, dual heating zones, rotating graphite substrate holder). By integrating the mechanisms of reaction gas flow, heat transfer, surface reactions between GaCl and NH_3_, and mass transport, the study systematically analyzes the flow field distribution, thermal field stability, and evolution law of the precursor concentration field within the reaction chamber over a pressure range of 91–141 kPa and quantifies the GaN deposition rate field and uniformity index on the substrate surface.

The core value of this research lies in filling the research gap in existing simulations regarding the “pressure-multi-physics coupling” correlation mechanism; clarifying the regulatory laws of pressure on growth rate (non-linear increase) and uniformity (stable at low pressure, deteriorated at high pressure); and establishing a “simulation—optimization—experiment” closed loop through subsequent experimental verification (high-quality 4 inch GaN single crystals were prepared based on the optimized pressure of 101–111 kPa, and XRD tests showed that the full width at half maximum (FWHM) of the (002) and (102) planes were both < 100 arcsec). This provides actionable theoretical and technological support for reducing experimental trial-and-error costs and achieving efficiency.

### 1.1. HVPE Growth Reaction Chamber

**Figure 1:**
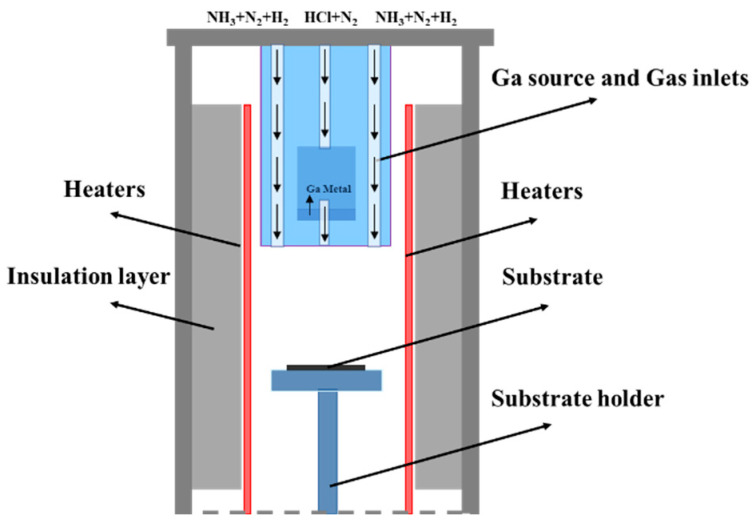
Simulation calculation model of HVPE vertical reaction chamber.

The structure of the vertical reaction chamber used for HVPE growth of GaN single crystals in the experiment is shown in Figure 1. The reaction chamber employs a cylindrical high-purity quartz tube (length 200 cm, radius 30 cm). A quartz tray is placed inside the quartz tube for holding the substrate. An embedded quartz tube (length 200 cm, radius 25 cm) serves as the gas inlet. A solid metallic gallium source with 99.999% purity is placed approximately 70 cm from the chamber inlet. Carrier gases such as H_2_/N_2_ and reaction gases such as NH_3_/HCl are introduced into the chamber through the embedded tube. The heating system primarily consists of a heat source matched to the quartz reaction chamber, approximately 150 cm long. It is divided into left and right vertical heating zones (each 15 cm wide), with a 15 cm constant temperature zone between the two zones. The exhaust filtration system collects waste composed of NH_4_Cl, GaCl, and Ga.

HVPE is a vapor-phase growth method. Its key mechanism involves an initial chlorination reaction in the low-temperature zone (~850 °C), potentially forming two Ga-Cl compounds: gallium monochloride (GaCl) and gallium trichloride (GaCl_3_).

Ga(l) + HCl(g) = GaCl(g) + H(a) (1a)

Ga(l) + 3HCl(g) = GaCl_3_(g) + 3H(a) (1b)

Under typical HVPE growth conditions, gallium monochloride is the predominant species [29,30,31]. N_2_ and H_2_ act as carrier gases, transporting it to the high-temperature zone (~1040 °C) where it reacts with NH_3_ on the substrate surface to form GaN at 1 atm pressure. The reaction is as follows:

GaCl(g) + NH_3_(g) = GaN(s) + HCl(g) + H_2_(g) (2)

Finally, unreacted gases and byproducts are purged from the reactor through a post-processing system to prevent contamination and improve film purity. Through the aforementioned process in the HVPE vertical reactor, GaN single crystals can be grown on the substrate surface.

The heating system of the HVPE vertical reaction chamber uses SiC material as the heating element, capable of rapid heating up to 1100 °C. The chamber outlet is connected to a vacuum mechanical pump, capable of achieving a pressure of about 10 Pa inside the reaction chamber. During the reaction, the gas lines are multi-channel; hydrogen chloride and ammonia gases are isolated from each other by nitrogen and hydrogen. Waste gas from the reaction between the gallium source and the substrate flows through the exhaust pipe into a tail gas treatment unit and is finally discharged. Table 2 lists the material parameters of various components in the HVPE growth reaction chamber, including material, density, thermal conductivity, isobaric specific heat capacity, and maximum operating temperature.

### 1.2. Finite Element Simulation Modeling

To accurately establish a numerical model for the GaN single crystal growth process, we used ANSYS Fluent finite element software to simulate the aforementioned vertical HVPE reaction chamber structure. Given the complexity of multi-physics field coupling distributions involved in the reaction process, the model was simplified as follows to enhance convergence efficiency and computational speed.

#### 1.2.1. Geometric Model Simplification

In the design and simulation of the HVPE reactor, geometric model simplification is crucial for balancing computational efficiency and physical accuracy. Table 3 summarizes the simplification strategies for various components, grounded in their physical characteristics.

#### 1.2.2. Gas Modeling

We assume that the gas mixture (GaCI/NH_3_/H_2_/N_2_) is an ideal gas, conforming to the equation of state (3):

(3) p=ρRT

where p: gas pressure; ρ: gas density; R: specific gas constant of the gas mixture (*R* = *R*_miversal_/M_mix_, M_mix_ is the molar mass of the mixture); *T*: absolute temperature. Intermolecular forces and volumes are neglected to simplify the calculations.

Our experimental conditions of ambient temperature and pressure were carried out, so the viscosity was chosen using the Sutherland model (4):

(4) $$\mu =\mu_{0}{\left(\frac{T}{T_{0}}\right)}^{\frac{3}{2}}\frac{T_{0}+S}{T+S}$$

where $\mu_{0}$: viscosity at reference temperature $T_{0}$; $T_{0}$: reference temperature (usually 273 K or 298 K); S: Sutherland’s constant (related to the gas type); *T* denotes the current absolute temperature when calculating viscosity; *μ*: gas viscosity; NH_3_: S = 370; HCl: S = 860 [32].

#### 1.2.3. Species Transport Equations

In the complex transport process in the HVPE reaction cavity, the spatial distribution and consumption rate of the reaction precursor (GaCl/NH_3_) directly determine the growth behavior of GaN, and its dynamic evolution needs to be accurately described by the species transport equation:

(5) $$\frac{\partial \left(\rho Y_{i}\right)}{\partial t}+\nabla \cdot \left(\rho \vec{u}Y_{i}\right)=\nabla \cdot \left(\rho D_{i}\nabla Y_{i}\right)+{\dot{\omega }}_{i}$$

where $Y_{i}$: mass fraction of component *i* (key components: GaCl, NH_3_, HCl); *ρ*: Total density of the gas; *t*: Time; *u*: Velocity vector of a gas; $D_{i}$: diffusion coefficient of component *i*; ${\dot{\omega }}_{i}$: source term of chemical reaction (mass production/consumption due to gas-phase reaction or surface deposition) of component *i*.

The binary diffusion coefficients *D_i_*, for gas pairs, required in Equation (5), were defined as temperature- and pressure-dependent. For common gases, values were sourced from standard references X, Y. For pairs involving GaCl, the coefficients were estimated using the Fuller–Schettler–Giddings method [X, Y] due to the lack of experimental data. The reference values and exponents for all critical pairs are provided in Supplementary Table S1 [33,34,35,36].

#### 1.2.4. Simplification of the Reaction Mechanism

In the numerical simulation of the HVPE reaction chamber, the physical nature of the gas flow is driven by the pressure gradient and the temperature gradient together: the pressure difference between the inlet and the outlet forms the main convection, while the density difference triggered by the thermal field inhomogeneity produces the buoyancy effect, and the two synergistically dominate the motion of the gas molecules. The process strictly follows the coupled multi-physics field governing equations, including the following: Transport of gallium atoms in vapor does not change their chemical state, and the GaCl synthesis reaction can only take place on the liquid gallium surface [30]. Furthermore, this model simplifies the gallium precursor into a single GaCl species, without considering the generation and transport of GaCl_3_. This simplification is based on extensive literature indicating GaCl as the dominant species within the temperature and pressure range studied herein [21,22,23], allowing the model to focus on the core physical processes of growth deposition. We acknowledge that the potential role of GaCl_3_ at higher pressures may exert minor effects on the finer details of growth kinetics. However, this does not alter the principal conclusion of this paper: pressure regulates growth behavior through partial pressure and transport effects. Future model refinements may consider incorporating more complex Ga-Cl vapor-phase chemical equilibria to achieve more precise predictions. Therefore, we simplify the gas-phase reaction to a surface deposition process, which is represented in the continuity equation by the mass source term:

(1) Continuity equation (conservation of mass).

(6) $$\frac{\partial \rho }{\partial t}+\nabla \cdot \left(\rho \vec{u}\right)={\dot{\omega }}_{dep}$$

where ${\dot{\omega }}_{dep}$: Mass source term (change in mass per unit volume per unit time) due to surface deposition; $\frac{\partial \rho }{\partial t}$: Unsteady term. Represents the rate of change of fluid density with respect to time at a fixed point in space; $\nabla \cdot \left(\rho \vec{u}\right)$: Convection term. Indicates that due to the flow of the fluid (velocity field $\vec{u}$) resulting in a net mass outflow rate; ∇⋅is the dispersion operator, which can be interpreted as a measure of whether a point is a “source” (outflow) or a “sink” (inflow).

(2) Equation of conservation of momentum (RANS framework)

(7) $$\rho \frac{D\vec{u}}{Dt}=-\nabla p+\nabla \cdot \left(\mu \nabla \vec{u}\right)+\rho \vec{g}$$

where $\frac{D\vec{u}}{Dt}$: material derivative $\left(\frac{\partial \vec{u}}{\partial t}+\vec{u}\cdot \nabla \vec{u}\right);-\nabla p$: pressure gradient force; $\nabla \cdot (\mu \nabla \vec{u})$: Viscous stress term (Newtonian fluids); $\rho \vec{g}$: gravitational term (in particle physics).

Turbulence model: k-ω SST model (compatible with high Reynolds number flow and low *Re* flow in the near-wall region). Combines the advantages of the k-ε model (high Reynolds number region) and the k-ω model (near-wall region). Suitable for full-flow simulation from the mainstream high Reynolds number region to the near-wall low Reynolds number boundary layer.

(3) The conservation of energy equation.

(8) $$\rho C_{p}\frac{DT}{Dt}=\nabla \cdot \left(k\nabla T\right)+{\dot{q}}_{\mathrm{chem}}$$

where $C_{p}$: constant-pressure specific heat capacity; k: heat conductivity; heat source term ${\dot{q}}_{\mathrm{chem}}$ from GaN deposition reaction (GaCl + NH_3_ → GaN + HCl): −230 kJ/mol [37].

The governing equations are presented within a general framework. However, for all simulations reported in this work, the laminar flow model was exclusively employed. This choice is rigorously justified by the low Reynolds number (Re < 2000) and the flow-stabilizing effect of the rotating susceptor, as detailed in Section 1.3, ensuring the model’s physical accuracy.

### 1.3. Boundary Condition

In this paper, the following boundary condition settings were made without affecting the experimental laws:

(1) Inlet: the inlet of the HVPE reaction chamber was fed with the carrier gas N_2_/H_2_ and the reaction gas NH_3_/HCl, respectively. The mass flow rate of the fed N_2_ is 1000 sccm; the mass flow rate of the H_2_ is 200 sccm; the mass flow rate of the fed NH3 is 150 sccm; and the mass flow rate of the HCl is 20 sccm.
(2) Cavity temperature: The reaction cavity wall was made of no-slip boundary ($u_{x}=u_{r}=0$) and adiabatic conditions ($\frac{\partial T}{\partial n}=0$), along with quartz surface emissivity ε = 0.8 for compatibility with radiative heat transfer models. The cavity has two pairs of heaters to control the high and low temperature zones The substrate surface is used as the core reaction boundary and is set to a fixed temperature (1073–1373 K) and rotational motion (10–100 rpm), and the surface reaction model is customized by UDF ($u_{x}=u_{r}=0$); the reactive nitrogen coverage θNH_3_ is calculated by the Langmuir adsorption equation and the GaN growth rate is correlated with the local GaCl concentration and temperature. It is important to note that the GaCl precursor is not introduced as a separate inlet stream. Instead, it is generated in situ from the reaction at the liquid gallium source surface, which is modeled as a reacting wall boundary with a species flux condition derived from the HCl consumption based on Equation (1a). The resulting GaCl concentration field upstream of the substrate is thus a solution outcome of the coupled transport and reaction simulation.
(3) Outlet: A mechanical vacuum pump on the right side of the chamber is responsible for controlling the constant pressure inside the chamber. The exhaust gases from the GaN source and substrate will flow through the exhaust pipe into the exhaust gas treatment device and finally out of the chamber. Fluid: There are two main forms of fluid flow in nature: one is universal turbulence and the other is the special case of laminar flow. The state of fluid flow in a circular tube is usually determined by the Reynolds number (also known as the Reynolds number), which is expressed in terms of *Re* as follows.

(9) $$Re=\frac{UD}{\nu }$$

In the equation, *U* represents the fluid velocity; ν denotes the kinematic viscosity; and *D* is the pipe diameter. Flow within the pipe is defined as laminar when the Reynolds number (*Re*) is less than 2000; otherwise, it is classified as turbulent. In this ANSYS Fluent simulation of the HVPE GaN growth reactor, the calculated Reynolds number (Re = 100–2000) under the dominant low-pressure process (5–50 kPa) is significantly below the critical value of 2000. Additionally, the rotating susceptor effectively suppresses turbulent vortices. Therefore, the laminar flow model was adopted to simplify computations.

Inlet V/III ratio (50:1) suppresses parasitic reactions; reduced outlet static pressure $p_{out}$ enhances diffusion uniformity; and surface reaction source term $S_{i}$ was validated via mass conservation equations (residuals < 10^−5^ kg/s), while thermal equilibrium equations correlate reaction exothermicity with heat dissipation power. All boundaries implement dynamic coupling through User-Defined Functions (UDFs), thus enabling precise prediction of growth behavior under pressure modulation.

### 1.4. Mesh Division and Solution Setup

In this paper, the effects of uniformity and deposition rate of GaN single crystals grown on the surface of sapphire substrate under different pressures are investigated, and the substrate is selected as a 4 inch (100 mm) c-face sapphire substrate. The remaining parameters such as gas flow rate (NH_3_, HCl, N_2_, H_2_), temperature difference between the high- and low-temperature zones (300 °C), and the distance between the air outlet and the substrate surface (200 mm) were kept consistent to control the variables.

In the ANSYS Fluent simulation meshing of the HVPE-GaN growth chamber, a MultiZone Sweep Mesh is used as the main topology. Axial layering: A structured hexahedral mesh is generated along the height direction of the cavity, and a 5° axisymmetric sector mesh is used for the rotational symmetric surface with periodic boundary conditions. Critical region encryption: 15 boundary layer meshes are set up on the substrate surface, with the first layer having a height of 0.01 mm (to ensure that the laminar model y^+^ ≈ 1) and a growth rate of 1.2. The nozzle–substrate gap is encrypted with an O-shaped cut block (minimum size of 0.2 mm) to capture the reactant concentration gradient gas inlet, which is bridged using a tetrahedral transition mesh. Full domain size control: 5 mm base size in the gas-phase main flow zone (balance of calculated efficiencies). Maximum size of 1 mm in the nozzle jet zone (to resolve high velocity shear flow); expansion ratio ≤ 1.2 in the near-wall region (to prevent mesh distortion). Mesh quality strictly follows the criteria of distortion < 0.7, aspect ratio < 20 (<50 in the nozzle zone), and orthogonality >30°. The reliability of the baseline grid size (1.58 million cells) was confirmed by grid-independence validation: the growth rate error for the coarser grid (0.52 million cells) was up to 4.1%, whereas the growth rate deviation between the baseline and the finer grid (4.12 million cells) was <0.5%, and the uniformity deviation converged to ±8.1%.

## 2. Results and Discussion

**Figure 2:**
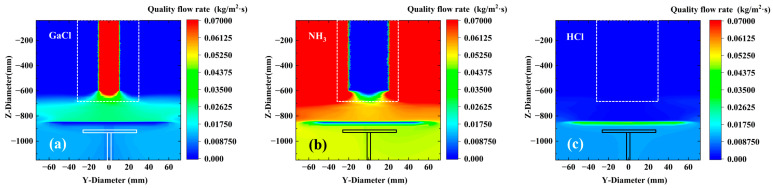
Mass flow rate distributions of main components in the growth model; (**a**–**c**) correspond to GaCl, NH_3_, and HCl, respectively.

In the experiment, our objective was to investigate the influence of pressure changes on the growth rate of gallium nitride single crystals. Shao et al. [38] found that the flow rate of GaCl carrier gas has a significant effect on the growth of GaN films by Hydride Vapor Phase Epitaxy (HVPE). Therefore, the settings for the carrier gas were kept consistent. As shown in Figure 2a,b, they correspond to the mass flow rates of GaCl and NH_3_ near the substrate, respectively. It can be observed from the figures that the concentration distribution of GaCl generally shows a pattern of being high in the middle and low on both sides. GaCl is typically injected into the reaction chamber at high speed through a central spray head, forming a high-momentum jet. The velocity along the jet centerline is the highest, and according to Bernoulli’s principle, the pressure in the high-speed region is lower, which attracts the surrounding fluid to converge toward the center. In contrast, the situation for NH_3_ is the opposite, exhibiting a low distribution in the middle and high distribution on both sides. We attribute this to the buoyancy effect (which enhances the edge aggregation of NH_3_). The molecular weight of NH_3_ (17 g/mol) is much lower than that of GaCl (105 g/mol). In a high-temperature reaction environment (>1000 °C), the light NH_3_ is driven by buoyancy to rise rapidly, while the heavy GaCl is delayed by gravity and sinks. This ultimately leads to the migration of NH3 toward the top/edges of the reaction chamber, while GaCl remains in the central bottom region (near the substrate). On the other hand, it may be due to local differences in reaction consumption (amplifying the asymmetry of the distribution). Because the central region of the substrate has the strongest thermal radiation, the surface reaction rate is the highest, thereby resulting in the maximum consumption of NH_3_.

**Figure 3:**
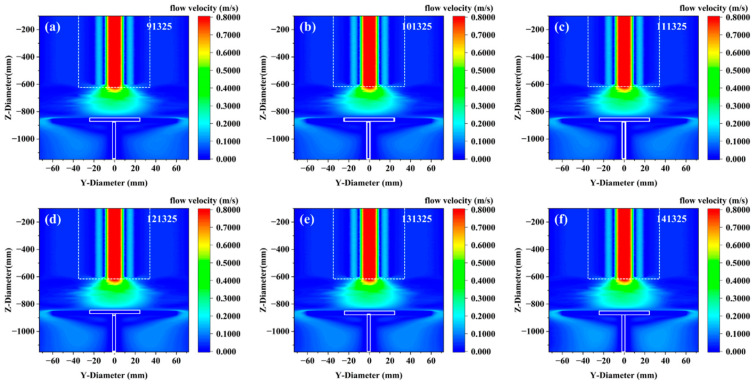
The flow rate changes in all gases in the growth model under different pressures; (**a**–**f**) correspond to pressures of 91,325–101,325 Pa.

Figure 3 shows the flow rate changes in all gases in the growth model under different pressures. It can be observed that pressure changes have essentially no effect on the overall gas flow distribution. The core mechanism behind the insignificant influence of pressure changes on the overall gas flow distribution lies in the flow similarity dominated by the Reynolds number (*Re*). When the reactor pressure increases, the gas density *ρ* increases proportionally. To maintain the same mass flow rate, the flow velocity *U* must decrease proportionally (U∝1/P). Since the Reynolds number formula Re=ρUD/μ remains constant ($\rho \cdot \left(1/P\right)\cdot P=$ constant), and the viscosity μ is independent of pressure in Sutherland’s model, with the geometric scale *D* unchanged, the *Re* number is strictly conserved. This similarity principle ensures that the ratio of inertial force to viscous force in the flow field, the flow state (laminar/turbulent), and the streamline structure all remain unchanged, leading to a high degree of consistency in the gas flow distribution under different pressures.

**Figure 4:**
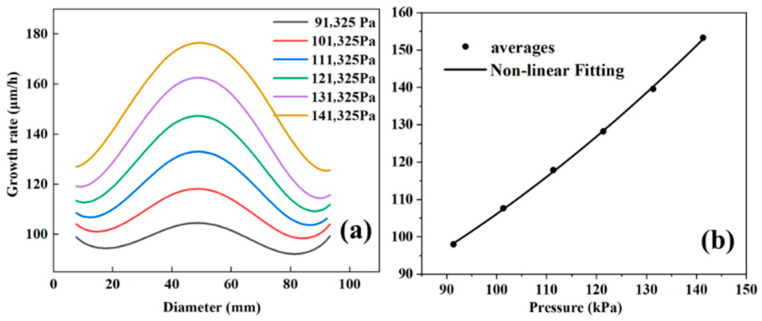
Growth rate of wafers under different pressures: (**a**) vertical distribution, (**b**) mean value variation.

The growth rate and growth uniformity of gallium nitride single crystals are discussed. As shown in Figure 4a, when the pressure increases from 91,325 Pa to 141,325 Pa, the growth rate also increases. The data points for this interval were integrated by polynomial fitting and found to be expressible in Equation (10):

(10) $$y=Intercept+B1*x^{\wedge }1+B2*x^{\wedge }2$$

Intercept denotes the intercept, which takes the value of 55.84541 + 11.71296; B1 and B2 are the coefficients of the primary and quadratic terms, respectively, which take the values of 0.05817 ± 0.20451 and 0.00445 ± 8.77327 × 10^−4^. The R^2^ of the curve is 0.99959.

Figure 4b shows the change in the average growth rate, which also exhibits the same trend. We argue that the core reason for the increased growth rate is the enhancement of surface chemical reaction kinetics. Since the growth rate is dominated by the reaction-controlled region ($R_{g}\propto P_{GaCl}$), and the feed molar fraction remains unchanged when the total pressure increases, the partial pressure of the key $P_{GaCl}$ is a nearly linear increase proportional to the total pressure ($P_{GaCl}\propto P$). This directly increases the adsorption concentration of GaCl on the substrate surface and the reaction collision frequency, driving the synchronous increase in the surface reaction rate of GaCl with NH_3_ (GaCl + NH_3_ → GaN + HCl). Eventually, the growth rate shows a nearly linear increase with the rise in pressure, which is consistent with the trend observed in Figure 4.

**Figure 5:**
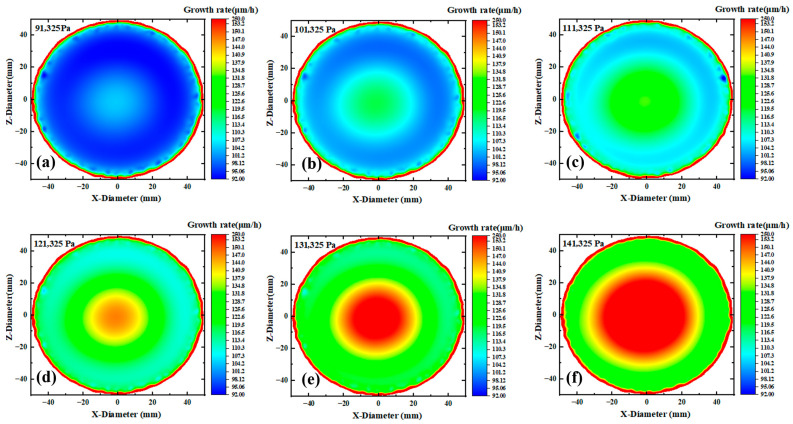
Schematic diagram of the growth rate of GaN at different pressures: (**a**–**f**) contour plots.

Figure 5a–f shows the growth rate contour maps of the substrate surface under pressures ranging from 91,325 to 141,325 Pa. On one hand, as the pressure increases, the substrate color transitions from purple to red, corresponding to the growth rate increase, which is consistent with the results in Figure 3. However, it can be observed that the growth rate at the edge approaches the maximum value under lower pressure, while this effect becomes less obvious at higher pressures. The phenomenon that increasing pressure weakens the relative advantage of edge growth rate originates from the spatial competition mechanism between buoyancy effect and diffusion limitation: Under low pressure (91.3 kPa), the lower gas density enhances the thermal buoyancy, driving the light NH_3_ to migrate toward the edge and form local enrichment. Meanwhile, the high diffusion coefficient accelerates reactant supply, making the edge growth rate approach the theoretical peak. Under high pressure (141.3 kPa), the increased gas density suppresses the buoyancy effect (Richardson number decreases by >30%), weakening the edge aggregation advantage of NH_3_. Meanwhile, the thickened boundary layer and reduced diffusion coefficient jointly exacerbate the edge transport limitation, while the central region dominated by reaction kinetics significantly improves the growth rate, leading to the weakened relative growth at the edge.

**Figure 6:**
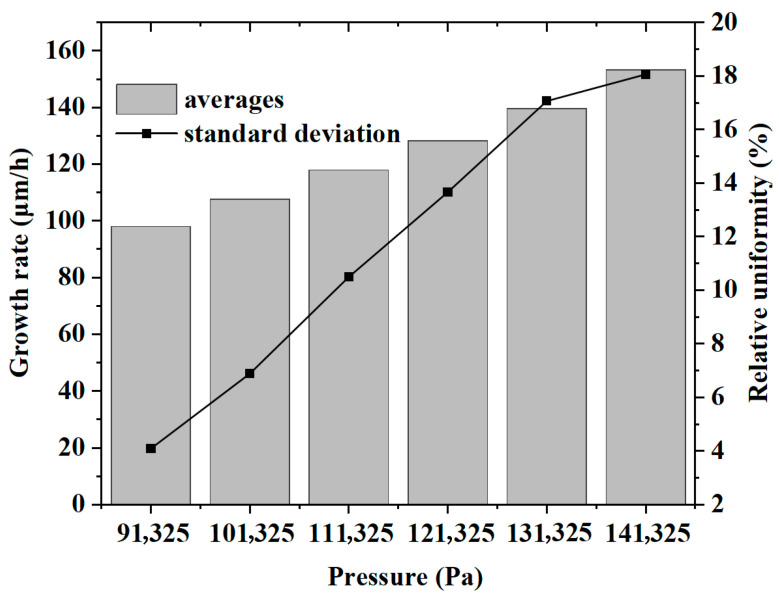
Mean and standard deviation of GaN growth rates under different pressures.

On the other hand, it can be observed that pressure changes have a significant impact on the growth uniformity of gallium nitride single crystals. As shown in Figure 5a–f, the growth rate of gallium nitride on the substrate exhibits significant differences. Additionally, Figure 6 clearly shows that as the pressure increases, both the mean growth rate and the standard deviation increase. However, the growth rate increases relatively uniformly, showing a nearly linear trend, while the standard deviation increases rapidly at lower pressures and slows down at higher pressures. The differential changes may be attributed to the dynamic balance mechanism between reaction control and transport control: In the low-pressure region (91–110 kPa), the mean growth rate increases by a nearly linear amount with pressure ($R_{avg}\propto P_{GaCl}\propto P$), but thermodynamic sensitivity is strong—minor temperature gradients are amplified by the Arrhenius exponent ($\Delta R/R\propto e^{E_{a}/RT}\cdot \Delta T$), causing rapid expansion of edge/center differences on the wafer and severe deterioration of uniformity. In the high-pressure region (>120 kPa), the thickened boundary layer ($\delta \propto P^{0.5}$) triggers a transport-dominated mechanism, reducing the temperature sensitivity of the growth rate ($\partial R/\partial T\propto P^{-0.5}$). Meanwhile, high pressure suppresses thermal buoyancy fluctuations (Richardson number $Ri\propto P^{-1}$ decreases by >40%), enhancing flow field stability. This makes the uniformity change tend to be gentle, forming a characteristic of a continuous nearly linear increase of the mean value and marginal diminishing improvement of uniformity.

**Figure 7:**
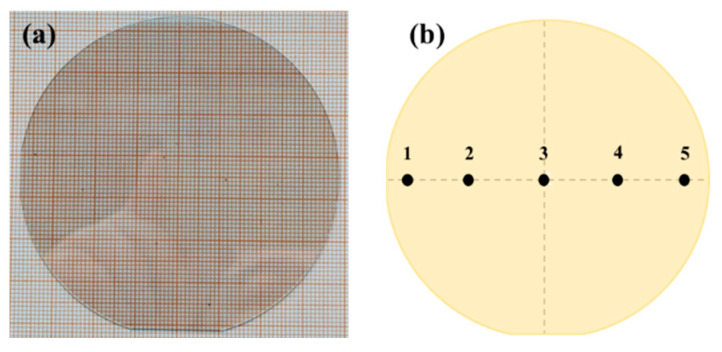
(**a**) Photograph of GaN single crystal substrate. (**b**) Selection of test points on the wafer. Numbers 1–5 denote the test points selected on the gallium nitride single crystal.

This study aims to verify the accuracy of the previous process simulation through experiments and optimize the GaN single crystal growth process based on the experimental results. The key optimization point of the experiment is to precisely control the reaction chamber pressure at a constant level of 101–111 kPa. This optimization measure effectively balances the contradiction between the crystal growth rate and wafer uniformity, thereby ensuring high-efficiency growth while achieving excellent consistency in wafer quality. Through the above process optimization, high-quality 4 inch gallium nitride single wafers were successfully prepared (as shown in Figure 7a). To rigorously evaluate their crystal quality, five points were selected for testing (as shown in Figure 7b). We conducted analyses using X-ray diffraction (XRD) and cathodoluminescence spectroscopy (CL), respectively.

As shown in Supplementary Figure S1, it can be observed that the full width at half maximum (FWHM) of XRD peaks for the (002) and (102) planes of GaN are both less than 100 arcsec, which can confirm its excellent crystal quality. Meanwhile, it can be found that the FWHM values of test points 1 and 5 are significantly higher than those of the other three points; that is, the crystallization quality at the edge is relatively poor. This is also consistent with the phenomenon of abnormal growth rate at the edge mentioned in the simulation above because an excessively high growth rate will inevitably lead to a decrease in crystal quality [39,40]. More details can be found in Table S2. As shown in Supplementary Figure S2, we evaluated the dislocations in different regions. It can be seen that the dislocation distribution is relatively uniform and essentially within the range of 10^6^–10^7^ cm^−2^, which belongs to a good dislocation density interval [41,42,43]. To conclusively identify the specific types of dislocations (threading mixed or edge type) and their nucleation sources, cross-sectional transmission electron microscopy (TEM) analysis of the wafer edge will be conducted in a future study. The analysis of XRD and CL characterization results shows that we have successfully obtained high-quality gallium nitride single crystal materials by optimizing key process parameters such as precise control of chamber pressure.

## 3. Conclusions

This work established a single-crystal growth furnace model for gallium nitride (GaN) on sapphire substrates using ANSYS Fluent finite element simulation, based on a vertical HVPE reactor. The influence of chamber pressure on growth rate and uniformity was investigated by varying the cavity pressure. Key findings include the following: Pressure changes have negligible impact on the overall flow field, likely due to Reynolds number-dominated flow similarity. Growth rate exhibited a nearly linear increase with pressure, while uniformity gradually deteriorated. In practical growth processes, maintaining the chamber pressure at an appropriate constant level (101–111 kPa) is recommended. This balance ensures both high growth rate and good wafer uniformity, which is particularly critical for growing large-size GaN single crystals. It should be noted that the experimental validation in this study focused on the optimal pressure window predicted by the simulations (101–111 kPa). Although this successfully validates the efficacy of the “simulation-optimization-experiment” closed loop in guiding process development, experimental validation at the boundary pressures would be a valuable extension in future work to further solidify the model’s universality.

## Figures and Tables

**Table 1:** Key properties of representative semiconductor materials from different generations.

Property	First Generation	Second Generation	Third Generation		
	Si	GaAs	SiC	GaN	AlN
Bandgap (eV)	1.1	1.4	3.2	3.4	6.1
Breakdown Field (MV/cm)	0.3	0.4	2.3	3.3	10–15
Electron Mobility (cm^2^/V·s)	1350	8500	400	1200	300
Thermal Conductivity (W/m·K)	150	50	400	130	285

**Table 2:** Material properties of various components in HVPE reactor.

Structure	Material	Density/(kg/m^−3^)	Thermal Conductivity /(W/m^−1^·K^−1^)	Constant Pressure Specific Heat Capacity/(J/kg^−1^·K^−1^)	Maximum Working Temperature/(°C)
Reaction chamber	Sapphire	2200	1.38	770	1200
Gas nozzle	SiC	1750	100	710	1600
Substrate base	Graphite	1800	100	710	1600
Substrate	Al_2_O_3_	3980	40	339	1800

**Table 3:** The simplification strategies for various components.

Structure	Streamlining Program	Physical Basis
Reaction chamber	Axisymmetric 2D model	Gas flow is symmetrical along the central axis
Ga source boat	Equivalent to a planar gallium source boundary	Ignore fluctuations in the metal gallium level
Swivel base	Moving Reference Frame (MRF) analog rotation	Replace actual rotation and maintain laminar flow characteristics
Gas nozzle	Ring inlet simplified to axial inlet	Ensure mass flow conservation

## Data Availability

The original contributions presented in the study are included in the article, further inquiries can be directed to the corresponding authors.

## Supplementary Materials

The following supporting information can be downloaded at: https://www.mdpi.com/article/10.3390/ma18214941/s1, Figure S1: XRD pattern of GaN. (a–e) correspond to test points 1–5 on side (002), (f–j) correspond to test points 1–5 on side (102), respectively; Table S1: Binary Diffusion Coefficient Parameters for Key Gas Pairs; Figure S2: CL spectrum of GaN. (a,b) correspond to test points 1 and 5. (c–e) correspond to test points 2–4; Table S2: Point-by-Point Comparison of Experimental and Simulated Results.
