# Supplementary material for: Simulation Study on the Effect of Growth Pressure on Growth Rate of GaN
Source: Materials (Basel). 2025 Oct 29;18(21):4941. doi: 10.3390/ma18214941 (PMC12610050; doi:10.3390/ma18214941)
Supplement: Supplementary file 1 [file materials-18-04941-s001.zip › materials-3896356-supplementary.pdf]

Supplementary material

Table S1. Binary Diffusion Coefficient Parameters for Key Gas Pairs.

Gas Pair (i-j)	$D_{0,ij}$ (cm^2/s)	T_0 (K)	Exponent n_{ij}	Source / Calculation Method
GaCl - N_2	0.150	273	1.75	Fuller-Schettler-Giddings method [33,34]
GaCl - H_2	0.410	273	1.75	Fuller-Schettler-Giddings method [33,34]
GaCl - NH_3	0.135	273	1.75	Fuller-Schettler-Giddings method [33,34]
GaCl - HCl	0.110	273	1.75	Fuller-Schettler-Giddings method [33,34]
NH_3 - H_2	0.760	273	1.75	Standard value from [35]
NH_3 - N_2	0.220	273	1.75	Standard value from [35]
HCl - H_2	0.670	273	1.75	Standard value from [35]
HCl - N_2	0.180	273	1.75	Standard value from [35]
H_2 - N_2	0.760	273	1.75	Well-established value from [35,36]

To quantitatively validate the simulation model, a direct point-by-point comparison between the experimental and simulated growth rates at the optimized pressure (~101 kPa) is provided in Table S2. The simulated growth rates show an excellent agreement with the experimental values, with a relative error of less than 8% across all five measurement points. This confirms that the model accurately captures both the spatial trend (center-low, edge-high) and the absolute magnitude of the growth rate. Furthermore, the experimentally observed degradation in XRD FWHM at the high-growth-rate edge points (1 and 5) aligns with the simulated prediction of heightened non-uniformity in these regions.

Table S2. Point-by-Point Comparison of Experimental and Simulated Results.

Measurement Point	Growth Rate (μm/h)		Relative Error	HRXRD FWHM (002) (arcsec)
	Simulated	Experimental		
1 (Edge)	169.9	180	+5.9%	76
2 (Mid-radius)	102.1	110.3	+8.0%	39
3 (Center)	118.0	126.5	+7.2%	43
4 (Mid-radius)	100.1	99.9	−2.0%	38
5	180.4	170.3	−6.7%	79

(Edge)

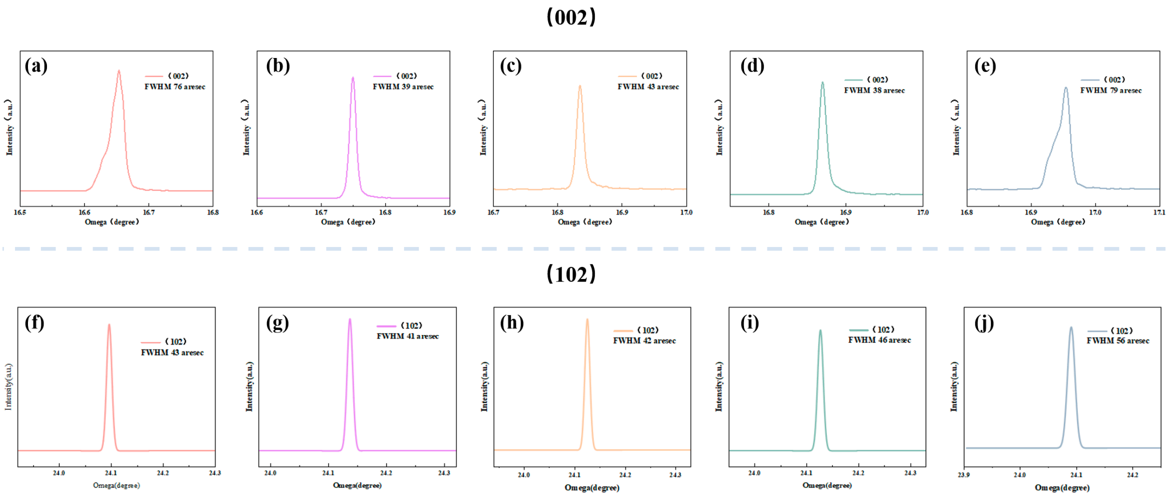

Figure S1. XRD pattern of GaN.(**a**) - (**e**) correspond to test points 1-5 on side (002), (**f**) - (**j**) correspond to test points 1-5 on side (102), respectively.

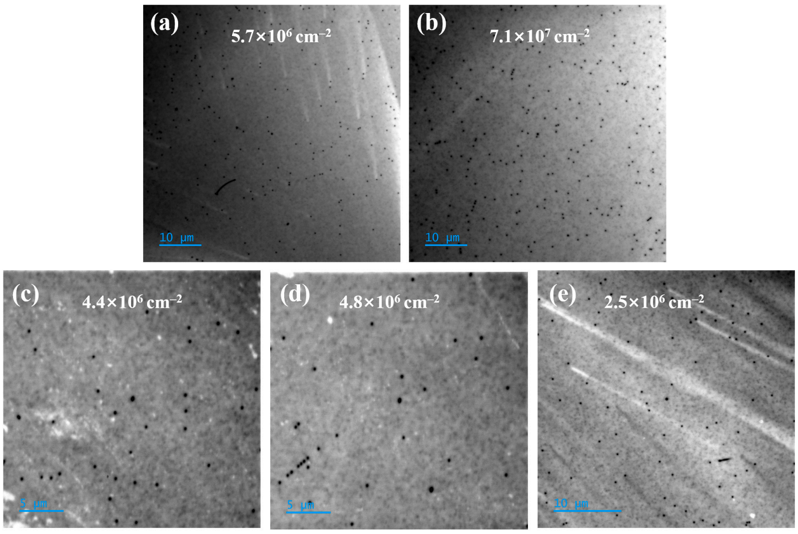

Figure S2. CL spectrum of GaN. (**a**) (**b**) correspond to test points 1 and 5. (**c**) - (**e**) correspond to test points 2-4.
